# Computational reactive–diffusive modeling for stratification and prognosis determination of patients with breast cancer receiving Olaparib

**DOI:** 10.1038/s41598-023-38760-z

**Published:** 2023-07-24

**Authors:** Francesco Schettini, Maria Valeria De Bonis, Carla Strina, Manuela Milani, Nicoletta Ziglioli, Sergio Aguggini, Ignazio Ciliberto, Carlo Azzini, Giuseppina Barbieri, Valeria Cervoni, Maria Rosa Cappelletti, Giuseppina Ferrero, Marco Ungari, Mariavittoria Locci, Ida Paris, Giovanni Scambia, Gianpaolo Ruocco, Daniele Generali

**Affiliations:** 1 Medical Oncology Department, Hospital Clinic of Barcelona, C. Villaroel 170, 08036 Barcelona, Spain; 2 Translational Genomics and Targeted Therapies in Solid Tumors, August Pi I Sunyer Biomedical Research Institute (IDIBAPS), Barcelona, Spain; 3 Faculty of Medicine, University of Barcelona, Barcelona, Spain; 4 Department for Sustainable Food Process, Università Cattolica del Sacro Cuore, Piacenza, Italy; 5 Department of Medicine, Surgery and Health Sciences, Cattinara Hospital, University of Trieste, Strada di Fiume 447, 34149 Trieste, Italy; 6 UO Anatomia Patologica ASST di Cremona, Cremona, Italy; 7 Department of Neuroscience, Reproductive Sciences and Dentistry, University of Naples Federico II, Naples, Italy; 8 Department of Woman and Child Health, Fondazione Policlinico Universitario A. Gemelli IRCCS, Rome, Italy; 9 Università Cattolica del Sacro Cuore, Rome, Italy; 10 Modeling and Prototyping Laboratory, College of Engineering, University of Basilicata, Potenza, Italy; 11 Multidisciplinary Unit of Breast Pathology and Translational Research, Cremona Hospital, Cremona, Italy

**Keywords:** Biomarkers, Medical research, Oncology, Mathematics and computing

## Abstract

Mathematical models based on partial differential equations (PDEs) can be exploited to handle clinical data with space/time dimensions, e.g. tumor growth challenged by neoadjuvant therapy. A model based on simplified assessment of tumor malignancy and pharmacodynamics efficiency was exercised to discover new metrics of patient prognosis in the OLTRE trial. We tested in a 17-patients cohort affected by early-stage triple negative breast cancer (TNBC) treated with 3 weeks of olaparib, the capability of a PDEs-based reactive–diffusive model of tumor growth to efficiently predict the response to olaparib in terms of SUV_max_ detected at ^18^FDG-PET/CT scan, by using specific terms to characterize tumor diffusion and proliferation. Computations were performed with COMSOL Multiphysics. Driving parameters governing the mathematical model were selected with Pearson's correlations. Discrepancies between actual and computed SUV_max_ values were assessed with Student’s t test and Wilcoxon rank sum test. The correlation between post-olaparib true and computed SUV_max_ was assessed with Pearson’s r and Spearman’s rho. After defining the proper mathematical assumptions, the nominal drug efficiency (ε_PD_) and tumor malignancy (*r*_c_) were computationally evaluated. The former parameter reflected the activity of olaparib on the tumor, while the latter represented the growth rate of metabolic activity as detected by SUV_max_. ε_PD_ was found to be directly dependent on basal tumor-infiltrating lymphocytes (TILs) and Ki67% and was detectable through proper linear regression functions according to TILs values, while *r*_c_ was represented by the baseline Ki67-to-TILs ratio. Predicted post-olaparib SUV*_max_ did not significantly differ from original post-olaparib SUV_max_ in the overall, gBRCA-mutant and gBRCA-wild-type subpopulations (*p* > 0.05 in all cases), showing strong positive correlation (r = 0.9 and rho = 0.9, *p* < 0.0001 both). A model of simplified tumor dynamics was exercised to effectively produce an upfront prediction of efficacy of 3-week neoadjuvant olaparib in terms of SUV_max_. Prospective evaluation in independent cohorts and correlation of these outcomes with more recognized efficacy endpoints is now warranted for model confirmation and tailoring of escalated/de-escalated therapeutic strategies for early-TNBC patients.

## Introduction

Breast cancer (BC) is the most common cancer in women and the most frequent cause of death by cancer in this sex^1^. Fortunately, in the last decades, new and escalated treatment strategies in early-stage disease have led to a substantial reduction in recurrence rates and improvements in survival^2,3^. However, treatment costs and toxicities have also increased substantially^4,5^. This has led to focus new research efforts in better personalizing therapeutic approaches, so to spare unnecessary toxicities and optimize BC care costs, as also advocated by the European society for medical oncology (ESMO) and the broader scientific community in recent years^6–8^. In this perspective, the development of tools capable of predicting tumor progression and response to novel therapies might help implementing therapeutic personalization and better identifying patients that might be spared long-term chemotherapy.

In the last few years, mathematical modeling has been entering the arena of oncological research in an attempt to predict spatial and temporal evolution of tumors transferring *in-silico* models to clinical research and practice^9,10^. Gompertzian and logistic mathematical models were first used to represent tumor cells’ growth and invasiveness and have been successively adopted in more sophisticated and complex models for tumor proliferation studies^11,12^. Accumulating evidence is showing that mathematical models based on partial differential equations (PDEs) are potentially exploitable to handle clinical data with spatial dimensions not solely depending on time; which is, for example, the case of tumor growth challenged by neoadjuvant therapy (NAT)^9,13–15^. The PDE approach based on reaction–diffusion models is often employed for cancer modeling. These models define the diffusion and proliferation of the various tumor components, including cancer cells, healthy cells, extracellular matrix etc. with specific mathematical formulas^16^. In this perspective, we preliminarily showed in a restricted cohort of 3 BC patients undergoing NAT, that a computational mass transfer modeling based on a set of PDEs applied at the tumor dynamics might represent a powerful in silico tool to virtualize tumor progression and predict tumor dynamics in response to therapy at the single-patient level^17^.

We hereby retrospectively applied our reactive–diffusive PDEs-based model to a wider BC patient cohort prospectively enrolled in a window-of-opportunity trial at our Institution^18^, to further test whether the combination of personalized diagnostic imaging and clinicopathological tumor/patient variables in mathematical modeling can accurately predict early-stage BC progression and its competition with a suitable NAT for a better patient-adapted planification of the therapeutic strategy.

## Methods

### Study population

This analysis was retrospectively performed on the BC patients enrolled within the OLTRE “window of opportunity” trial (NCT02681562) with available data for the mathematical modeling. Within the OLTRE study, conducted at the ASST Cremona between 2016 and 2019, treatment-naïve patients with locally advanced non-metastatic HER2-negative BC, with or without a germline *BRCA1/2* (gBRCA) mutation, received the PARP inhibitor (PARPi) olaparib at a dose of 300 mg orally for 21 consecutive days, before starting the standard neoadjuvant chemotherapy (CT)^18^. The main objective of the trial was to explore the biological effects of a short course of olaparib, especially in locally advanced triple negative BC (TNBC) independently of the gBRCA status.

All patients underwent a ^18^FDG-PET/CT scan at baseline and after 3 weeks of olaparib ± 3 days; and clinical assessments were conducted at baseline and every 3 weeks ± 3 days. Clinical responses were evaluated through physical exam with caliper and assessed according to RECIST1.1 criteria ^18,19^. The same operator performed all physical examinations pre/post olaparib to identify clinical responders (complete response + partial response [CR/PR]) and non-responders (stable disease + progressive disease [SD/PD]). ^18^FDG-PET/CT was used to detect radiometabolic responsiveness to olaparib by taking into account baseline and post-olaparib maximum standard uptake value (SUV_max_) values for the primary lesion. The same radiologist evaluated all PET/CT responses. Full study details are reported elsewhere^18^. Only TNBC patients were included in the present analysis.

### Study hypotheses and objectives

The main hypothesis behind this sub-analysis of the OLTRE trial was to test an *in-silico* reactive-diffuse model based on PDEs to predict the BC metabolic response to NAT with olaparib alone, as detected by ^18^FDG-PET/CT in terms of SUV_max_ after 3 weeks of neoadjuvant olaparib. To differentiate between actual and predicted SUV_max_ values in silico, we will refer to the latter as SUV*_max_. As a consequence, for the purpose of the present study, tumor dimensional assessments were not considered for the development of our mathematical model.

### Study procedures: theoretical premises

From the engineering point of view, BC is a single-phase (solid) biomaterial, featuring sharp boundaries delineating the cancer cells population Ø_c_, that grows and invades a region of interest (ROI)^10^. During NAT, a given mass rate of olaparib Ø_d_ was administered. When Ø_c_ represents an index of metabolic activity, its integration in the ROI can be compared with the measurement of SUV_max_ by ^18^FDG-PET/CT and a Gompertzian logistic function can be employed to describe its change rate, as reported in equation Eq. (1), as we and others preliminarily demonstrated^11,20–22^.

1 $$\frac{{{\text{d}}\phi_{c} }}{{{\text{d}}t}} = - r_{c} \phi_{c} \ln \left( {\frac{{\phi_{c} }}{K}} \right)$$

**Figure 1:**
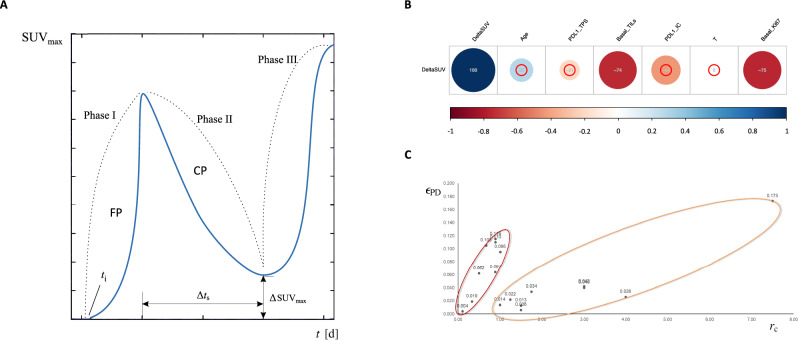
Key methodological steps of the mathematical model. (**A**): Gompertzian curve representing tumor metabolic activity in terms of SUV_max_ in different cancer growth phases; (**B**): Pearson’s correlations among SUV_max_ modifications under olaparib and baseline clinicopathological parameters of interest. (**C**): Nominal personalized olaparib efficiency ε_PD_ versus nominal personalized breast cancer malignancy *r*_c_; t, time; delta, variation; i, initial; FP, free tumor proliferation phase (Phase I); CP, challenged tumor proliferation phase (Phase II); T, primary tumor size; TPS, tumor proportion score; IC, immune cells; TILs, tumor-infiltrating lymphocytes; SUV, standard uptake value. In panel B grey numbers are Pearsons’ coefficients. The more positive the correlations, the darker the blue circles, while the more negative the correlations, the darker the red circles. The peripheral red circles identify non-significant correlations.

In the equation, *r*_c_ represents the nominal personalized biological conversion rate depending on the nanoscale (genomics) and the microscale (cell signals/molecular biology), such as BC invasiveness, aggressiveness or malignancy. In our analysis, *r*_c_ reflects the growth rate of metabolic activity as detected by SUV_max_, surrogate of BC malignancy. The 1/*r*_c_ is a timescale constant, representing the growth rate of Ø_c_. The variable *t* stands for time, while the parameter K is an arbitrary constant representing a surrogate of the carrying capacity of the biological matrix. Namely, the limiting nutrients for the onset of the metabolic conversion in the confined space where the BC lesion is detected. The value of K is taken such that the sigmoid function described by Eq. (1) approaches its future asymptote very gradually. Following the Gompertzian function reported in Eq. (1), the tumor growth dynamics can be subdivided into three phases, represented in Fig. 1A. A Phase I of free tumor proliferation (FP), when the lesion starts growing from an unknown time (*t*_i_) in the past, until it is first diagnosed at time 0 (*t*_0_). The following Phase II is also called challenged proliferation (CP), representing the time when a drug is administered and, if effective, impairs tumor growth/metabolic activity. Usually, a phase III of further tumor growth/increase in metabolic activity is observed anytime a treatment is ceased or resistance is developed. Surgical resection or a treatment change interrupts this phase. Since we aimed at predicting the tumor metabolic activity as detected by ^18^FDG-PET/CT in terms of SUV_max_ after 3-week neoadjuvant olaparib alone, we considered as *t*0 the time of baseline SUV_max_ detection.

### Governing equations and model assumptions

Two PDEs for reaction–diffusion were used in our mathematic model, represented by Eqs. (2) and (3)^20^.

2 $$\frac{{\partial \phi_{c} }}{\partial t} = \nabla \cdot \left[ {D_{c} \nabla \phi_{c} } \right] + R_{c}$$

3 $$\frac{{\partial \phi_{d} }}{\partial t} = \nabla \cdot \left[ {D_{d} \nabla \phi_{d} } \right] + R_{d}$$

Equation (2) represents the tumoral biomass transport, in terms of dimensionless metabolic activity indicator, while Eq. (3) the drug transport, in terms of olaparib concentration. The source terms *R*_c_ and *R*_d_ are defined by the following equations:

$$\begin{gathered} R_{c} = - r_{c} \phi_{c} \ln \left( {\frac{{\phi_{c} }}{K}} \right) - \epsilon_{{{\text{PD}}}} \phi_{{\text{d}}} \hfill \\ R_{{\text{d}}} = f\left( t \right) - \epsilon_{{{\text{PK}}}} \phi_{{\text{d}}} \hfill \\ \end{gathered}$$

Importantly, the following assumptions were taken: (1) the physical and functional lag consisting in multiple compartments mediating drug delivery to the tumor lesion was not taken into account, since the effect of mediating compartments would be approximately the same for each single patient and the study had an overall a short time span (3 weeks of olaparib administration)^23^; (2) a detailed action of the extra-cellular matrix (ECM) was not considered in this model. However, the crosstalk between cancer-associated fibroblasts (CAFs) and tumor cells was taken into account in the bulk effect of the effective diffusion coefficient of tumoral biomass, *D*_c_ in Eq. (2)^24,25^; (3) *D*_d_ in Eq. (3) represented the diffusion coefficients for the drug, i.e. olaparib. For simplicity, we considered the same *D* values for all patients enrolled into the study (i.e. *D*_c_ = 1^e−13^ m^2^/s; *D*_d_ = 1e^−5^ m^2^/s)^26^.

Moreover, to fully understand the above-mentioned equations, the following definitions were adopted:

- ε_PD_: nominal drug efficiency, or aggregated personalized pharmacodynamic (PD) behavior of the drug (i.e. olaparib). It represents the effect of the drug on the tumor in terms of either tumor shrinkage or, in this study, SUV_max_ reduction;
- *f*(*t*): the indicator of therapy regimen for the administered drug, equal to 1 during the entire neoadjuvant treatment duration. Therefore, the drug concentration in the patient blood was assumed to be constant;
- ε_PK_: the known effect of the clearance, or pharmacokinetic (PK) behavior for the administered olaparib, based on the available drug specifications (i.e. olaparib nominal plasma clearance: 7 l/h^27,28^). In other words, ε_PK_ brings purposely the PK effect into the PDE representing the distribution of drug concentration. To this end, the drug’s nominal plasma clearance (converted in m^3^/s) must be multiplied for the patient’s nominal density (in kg/m^3^, conventionally assumed equal to water) then divided for the patient’s mass (in kg), in order to obtain ε_PK_’s desired unit of 1/s, to reach unit consistency in the PDE. This parameter is essential to calculate *R*_d_, as previously reported.

Due to the integration of the PDEs system described by Eqs. (2) and (3), the Ø_c_ and Ø_d_ evolve in space and time. The space integration is performed in the available breast volume, while the variables progress over time (*t*), starting from the *in-silico* starting time of BC lesion (*t*_i_) to the end of olaparib NAT.

### Study procedures: analysis

The system of equations applied to the available breast volume, Eqs. (2–3), supplemented by source term *R*_c_ and *R*_d_ definitions equations, along with their initial and boundary conditions previously described, were integrated with the Finite Element Method, by using the COMSOL Multiphysics platform^29^. An unstructured meshing technique was used, yielding for a homogeneous tetrahedral element grid. After a grid independency test, a final mesh of approximately 10.000 elements was employed, to optimize result accuracy and computational times^17^. Direct solver PARDISO was employed as the algebraic engine, while the BDF method was applied for the temporal dependence^17^. Execution durations, for each patient, did not exceed 30 min on a Pentium® Xeon server (Windows® 10 [Microsoft, Redmont, WA, USA], Eightcore-32N at 2.4 GHz, 128 GB RAM) running in serial mode.

Population characteristics were assessed through standard descriptive statistics and variations in mean for main pathological variables pre/post olaparib were assessed through Students’ t-test for paired samples. The correlation of the main clinicopathological features (i.e. Ki67%, tumor-infiltrating lymphocytes [TILs] %, age in years, tumor size in mm, PD-L1 TPS and IC %) with the difference between post- and pre-olaparib SUV_max_ values, were used to identify the parameters better correlating with tumor malignancy *r*_c_ to provide the necessary estimations within the mathematical model. Finally, the post-olaparib SUV_max_ and SUV*_max_ were compared with both parametric (unpaired Student’s t-test) and non-parametric (Wilcoxon rank sum test) tests to assess the global mean/median difference between the actual and predicted parameter of interest. The correlation between true and computed post-olaparib SUV*_max_ values was assessed with Pearson’s r and Spearman’s rho. Significance was set at *p* < 0.05. R vers. 3.6.1 for MacOSX was used for statistical analysis.

### Ethical approval

The OLTRE trial (NCT02681562) was conducted in accordance with the Declaration of Helsinki, the Good Clinical Practice principles and all local regulations. The study obtained the approval of the ethical committee of the ASST of Cremona Hospital (IRB Approval 09/09/2015 n.21741/2015) and all participants provided written informed consent for participation.

## Results

The population of the OLTRE trial has been already described elsewhere^18^. For the purpose of the present study, 17/35 patients presented all the sufficient data to be included (i.e. all pre/post SUV_max_ values and baseline clinicopathological features). All selected patients were affected by locally advanced TNBC, with 5 (29.4%) carrying a gBRCA mutation, while the remaining 12 (70.6%) were gBRCA-wild type. Main population features are reported in Table 1. All patients underwent 3 weeks of olaparib according to study protocol and obtained a significant reduction in tumor dimension (mean of the difference in lesion mm: 10.21, 95% confidence interval [CI]: 5.00–15–42, *p* < 0.001) and metabolic activity (mean of the difference in SUV_max_: 4.33, 95% CI 1.54–7.12, *p* = 0.004). We retrospectively applied our computational reactive–diffusive modeling approach on this subset of 17 patients to predict SUV_max_ response. An inspection on the analytical structure of the model revealed that, once applied the known *f*(*t*) and ε_PK_ for the specific patient, the progress in the FP phase (Fig. 1A) depended on the previously mentioned *t*_i_ and *r*_c_ parameters. Therefore, we firstly performed multiple Pearson correlation analyses to identify the clinicopathological factors potentially associated with SUV_max_ modifications. We noted the SUV_max_ reduction obtained with olaparib was significantly inversely correlated with baseline Ki67 and TILs levels (r = − 0.75 and r = − 0.74 , respectively, *p* < 0.001 both) only (Fig. 1B). Hence, tumor malignancy, identified with the parameter *r*_c_ (i.e. SUV_max_ growth rate) was subsequently defined as Ki67/TILs ratio. This mathematical definition was based on the evidences showing in early-stage TNBC that higher values were associated with better survival outcomes^30–32^. We normalized Ki67 and TILs values in order to obtain a value range comprised between 0 and 1. Then, the lesion starting time *t*i was tweaked in each case to closely match each measured baseline SUV_max_, at the end of the FP phase. Next, for each single patient the models were iteratively run by manually-adjusting the nominal personalized olaparib efficiency (ε_PD_) in order to come up, in the subsequent CP phase, with minimal relative deviations in the computed post-olaparib SUV*_max_ volumes, with respect to the corresponding actual post-olaparib SUV_max_ measurements. Results are summarized in Table 2.

**Table 1:** Patients demographics.

CHARACTERISTICS	BASELINE		POST-OLAPARIB		p*
	N	%	N	%	
	17	100.0	17	100.0	
Age (years)					
Mean	61.4	–	–	–	–
SD	±	–	–	–	
*Total*	17	100.0	–	–	
Ki67 (%)					
mean	51	–	52	–	0.437
SD	± 25.7	–	± 27.9	–	
*Total*	17	100.0	16	94.1	
TILs (%)					
mean	54.7	–	54.3	–	0.577
SD	± 36.1	–	± 34.6	–	
*Total*	17	100.0	14	82.4	
T (mm)					
mean	40.1	–	29.4	–	< 0.001
SD	± 16.8	–	± 15.0	–	
*Total*	17	–	17	–	
SUV_max_					
mean	8.6	–	4.2	–	0.004
SD	± 5.1	–	± 2.5	–	
*Total*	17	100.0	17	100.0	
PD-L1 TPS					
Positive	9	52.9	5	55.6	0.572
Negative	8	47.1	4	44.4	
*Total*	17	100.0	9	52.9	
PD-L1 IC					
Positive	11	64.7	8	88.9	0.570
Negative	6	35.3	1	11.1	
*Total*	17	100.0	9	52.9	
gBRCA					
Mutant	5	29.4	–	–	
Wild-type	12	70.6	–	–	
*Total*	17	100.0	–	–	

*SD* standard deviation, *TILs* tumor-infiltrating lymphocytes, *T* maximum diameter of the primary tumor as measured by calliper, *SUV* standard uptake volume, *PD-L1 TPS* PD-L1 positivity assessed according to the tumor proportion score^16^, *PD-L1 IC* PD-L1 positivity assessed on immune cells^16^, *gBRCA* germline *BRCA1/2*.

*p values from Students’ t-tests for paired samples.

**Table 2:** Detailed values of the driving parameters of the mathematical model, with clinical and computed SUV_max_.

Patient	*t*_i_	*r*_c_	ε_PD_	Baseline SUV_max_	Post-olaparib SUV_max_	Baseline SUV*_max_	Post-olaparib SUV*_max_
1	814	3.33E−08	1.90E−02	6.5	3.7	6.51	3.7
2	202	1.50E−07	6.20E−03	3.8	4.8	3.8	4.79
3	43	7.50E−07	1.73E−01	6	2.1	6.16	2.08
4	108	3.00E−07	4.00E−02	10.3	9.6	10.46	9.63
5	180	1.80E−07	3.40E−02	6	4.3	6.08	4.34
6	590	5.00E−08	6.20E−02	11.6	2.9	11.61	2.89
7	252	1.25E−07	2.20E−02	9.8	7.9	9.74	7.8
8	80	4.00E−07	2.60E−02	1.9	2.9	1.92	2.91
9	2147	1.11E−08	4.00E−03	8	6.5	8.12	6.5
10	326	8.89E−08	6.40E−02	3.8	1	3.84	0.99
11	312	1.00E−07	9.50E−02	9.3	1.5	9.3	1.49
12	291	1.00E−07	1.40E−02	3.5	3	3.48	3.04
13	353	8.89E−08	1.15E−01	12.9	1.3	12.96	1.28
14	106	3.00E−07	4.20E−02	7.3	6.3	7.19	6.38
15	357	8.89E−08	1.10E−01	21.9	2.7	21.74	2.69
16	208	1.50E−07	1.30E−02	6.5	6.8	6.51	6.87
17	461	6.67E−08	1.05E−01	17	3.5	16.98	3.46

*t*_i_, lesion starting time; *r*_c_, biological conversion rate. In this case each r_c_ is the results of Ki67/TILs; ε_PD_, olaparib efficiency (effect of the drug on the body); SUV_max_, actual maximum standard uptake volume measured by ^18^FDG-PET/CT; SUV*_max_, maximum standard uptake volume calculated by the computational model.

At this point, we had a very good reproduction of each clinical case, but no actual potential of prospective evaluation, yet. We then plotted ε_PD_ against *r*_c_ (i.e. Ki67/TILs ratio) discovering that two sub-cohorts appeared neatly depending on the range of normalized TILs level (Fig. 1C**)**. The patient normalized Ki67 and TILs values could be grouped along two different interpolating curves based on the following functions:

1. for TILs level below the value of 0.5: ε_PD_ = 0.1227 · *r*_c_^1.5184^, with R^2^ = 0.9277
2. for TILs level above the value of 0.5: ε_PD_ = 0.004 · *r*_c_^2 ^+ 0.0105 · *r*_c_ + 0.0252, with R^2^ = 0.9448

Hence, with ε_PD_ expressed with the above dependences, all of the driving parameters in Eqs. (2–3) were identified, and the model could be solved in a full prospective mode, with its solution depending on the available baseline SUV_max_ and baseline Ki67 and TILs levels. Consequently, in a second computational stage the models were iteratively run again, by keeping the same optimized values for *t*_i_ and *r*_c_ reported in Table 1. The computed SUV*_max_, along with the relative deviations with respect to the corresponding true SUV _max_ measured by PET, are listed in Table 3.

**Table 3:** Values of εPD for each patient, with related SUV*_max_ and their absolute deviations with respect to the clinical measurements of post-olaparib SUV_max_.

Patient	εPD	SUV*_max_ Post	SUV_max_ Post-SUV*_max_ Post
1	2.31E−02	3.28	0.42
2	1.85E−02	3.71	1.09
3	1.71E−01	2.13	0.03
4	2.97E−02	11.53	1.93
5	1.91E−02	6.27	1.97
6	4.28E−02	4.5	1.6
7	1.83E−02	8.46	0.56
8	4.72E−02	2.01	0.89
9	4.36E−03	6.37	0.13
10	1.03E−01	0.33	0.67
11	1.23E−01	0.75	0.75
12	1.87E−02	2.83	0.17
13	1.03E−01	1.72	0.42
14	2.97E−02	7.92	1.62
15	1.03E−01	3.08	0.38
16	1.85E−02	6.36	0.44
17	6.63E−02	3.39	0.11

SUV*_max_, predicted SUV_max_ values; εPD, olaparib efficiency (effect of the drug on the body); SUV, standard uptake volume.

The prediction quality was adequate, with computed post-olaparib SUV*_max_ differing of < 1 unit with respect to the actual post-olaparib SUV_max_ for 12 (70.6%) patients and in the range of 1–2 units for the remaining 5 (29.4%), independently from gBRCA status. The correlation between post-olaparib SUV_max_ and computed SUV*_max_ was positive and very high, according to parametric and non-parametric methods, as well (Pearson’s r = 0.95, *p* < 0.0001, Spearman’s rho = 0.93, *p* < 0.0001). Importantly, the numerical difference between post-olaparib SUV_max_ and SUV*_max_ was not statistically significant by using both parametric (*p* = 0.813, *p* = 0.866 and *p* = 0.856) and non-parametric (*p* = 0.945, *p* = 0.841 and *p* = 0.977) statistics for the overall, gBRCA-mutant and gBRCA-wild-type populations, respectively.

**Figure 2:**
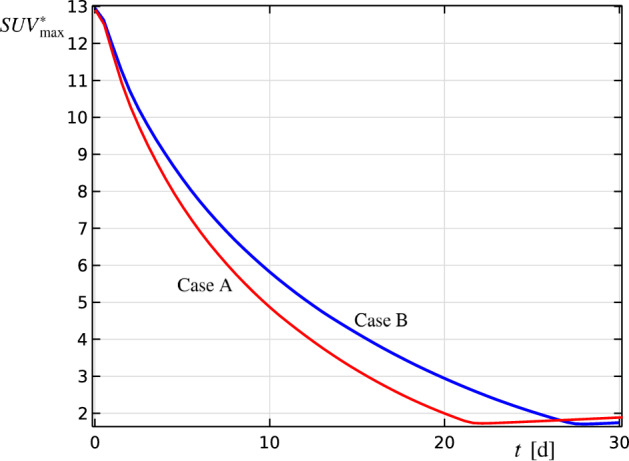
Virtual scenario of different pharmacodynamic efficiency and olaparib duration. Computed SUV*_max_ progressing with time for a real and a fictious case, having a different nominal personalized olaparib efficiency ε_PD_ and a different NAT duration. The progress is reported limited to Phase II of challenged proliferation. Case A: results from patient n.13 of our cohort; Case B: virtual case of patient n.13 with 20% decrement of ε_PD_ and a total NAT duration of 27 days instead of 21; *t*, time; d, days.

Finally, we exercised the model in a virtual scenario of different pharmacodynamic efficiency (ε_PD_) and olaparib duration, for a randomly selected patient of our cohort (patient n.13), with the objective of bringing out the non-linear relationship between the predicted outcome (SUV*_max_) and time. As observable in Fig. 2, the progress of the tumor computed SUV*_max_ for patient n.13 was compared, with the variable values leading to the results reported in Table 3 (Case A), to a virtual case represented by the same patient with a 20% decrement of ε_PD_ and an increase in 6 days of olaparib duration (Case B).

We observed that in case of variation of both response to NAT and NAT duration, it was again possible to achieve the same quantitative reduction in predicted SUV_max_ (i.e. SUV*_max_ = 1.72) by stretching the NAT duration (Fig. 2). Such results were obtained by executing the model iteratively, until the same original volume of Case A was achieved. It is confirmed therefore the results obtainable from the proposed model, through its non-trivial solution, are strongly intertwined and non-linear in nature, and the model presents the flexibility and adaptability to potentially tailor in silico an entire therapeutic strategy at the patient level.

## Discussion

We tested, in a cohort of 17 patients affected by early-stage TNBC treated with 3 weeks of olaparib in a “window of opportunity” trial, a mass transfer PDEs-based reactive–diffusive mathematical model of tumor growth which might be capable of efficiently predicting the response to olaparib in terms of SUV_max_ detected at ^18^FDG-PET/CT scan.

The model showed, without any preliminary assumption, the effective pharmacodynamic efficiency of olaparib was strongly dependent on basal TILs level and SUV_max_ growth rate. The latter was represented by a mathematical parameter that in our case was directly dependent on Ki67 expression and TILs count. By knowing the basal SUV_max_, Ki67% and TILs levels it would be possible with this approach to predict with a very small margin of error the SUV_max_ change after 3 weeks of olaparib. Although, this is not standard of care, it opens up to the possibility of early-detecting a drug efficacy (olaparib in this case) and investigating the personalization of escalated or de-escalated therapeutic strategies in early-stage TNBC in future research.

The need for biomarkers to guide escalated/de-escalated and more personalized therapeutic approaches is a hot topic in current Oncology^33,34^. Moreover, TNBC are historically a subgroup of breast tumors with poor prognosis and lack of biomarkers for effective personalized therapeutic approaches^35,36^. The most notable and very recent (for their therapeutic implications) exceptions are the determination of gBRCA status, in both adjuvant and metastatic setting, to decide whether or not to prescribe PARPi and the assessment of PD-L1 levels/positivity for the prescription of 1^st^-line immunotherapy + chemotherapy in metastatic disease^37–39^. However, the main issues with biomarker discovery research include: (1) higher difficulty related to lack of funding or difficult access to data from clinical trials, (2) regulators are less prone/used to approve prognostic and predictive biomarkers than novel therapeutic options, (3) the high number of false discoveries, (4) lack of reproducibility or complex/inviable implementation in clinical practice^40–42^. This translates into a slow and defective transfer of knowledge from the laboratory to the clinical research and/or practice scenario.

Noteworthy, the evaluation of Ki67 is an already established prognostic biomarker in BC, which expression is strongly associated to tumor proliferation and growth^43^. Higher levels are usually associated to higher tumor aggressiveness and worse prognosis, although in TNBC there is no established cut-off to define high vs. low Ki67 levels, differently from hormone receptor-positive BC^35,43,44^. Nevertheless, a recent meta-analysis of 35 independent studies (~ 8000 patients with resected TNBC) suggested that a cut-off of 40% would be associated with higher recurrence risk and mortality^45^.

The morphological evaluation of TILs in BC has gained attention in the last few years, when preliminary evidences started to show a potential prognostic and predictive role, especially in TNBC and HER2-positive BC^46^. A recent retrospective analysis on more than 2000 patients showed a clear favorable prognostic role for higher TILs levels (≥ 30%) in early-stage TNBC, independently from main clinicopathological factors^30^. This evidence adds to the strong independent prognostic role showed by TILs in residual disease after neoadjuvant chemotherapy^47^ and a retrospective analysis where higher TILs were found to be independently associated to multiple survival endpoints in patients from an old cohort not treated with (neo)adjuvant chemotherapy. In the same study, stage I tumors with TILs ≥ 30% showed a 5-year overall survival of 98%^48^. These evidences assure our mathematical model was built on biologically and clinically meaningful parameters. Furthermore, our model might be a powerful tool for the personalization of BC care not necessarily requiring the detection of novel costly biomarkers.

There are several limitations that will have to be overcome to promote the implementation of this mathematical framework in the clinical research and practice scenarios. First of all, we had the possibility to study our mathematical model in a cohort of 17 patients, which is more than what is usually done in this research field, where modelling frameworks based on single patients are the norm^13,17,21^. However, wider cohorts are required to validate our findings. Secondly, olaparib is still not approved in the neoadjuvant setting, nor in gBRCA-wild type TNBC. Nevertheless, PARPi showed activity and efficacy in several solid tumors also beyond gBRCA mutational status^38^ and the model performed quite well in both gBRCA-wild-type and mutant patients. Moreover, another PARPi, namely talazoparib, already showed promising neoadjuvant efficacy in gBRCA-mutant TNBC^49^ and the same olaparib is currently under evaluation in a phase II study in monotherapy or in combination with the immune-checkpoint inhibitor durvalumab in early-stage HER2-negative BC with either a germline or somatic *BRCA1/2* mutations (OlimpiaN, ClinicalTrials.gov identifier: NCT05498155). In addition, also the PARPi niraparib recently showed in a pilot study, a high tumor response (90.5%) on MRI along with high pCR rate (40.0%) in gBRCA-mutant HER2-negative breast cancer patients who received it as monotherapy in the neoadjuvant setting^50^. This means that PARPi have the potential to become a neoadjuvant therapeutic option for TNBC in the next future and the mathematical model hereby tested might be envisioned as an in silico predictor of response for further upfront implementation of escalated (e.g. the addition of immunotherapy and/or addition of posterior chemotherapy) or de-escalated strategies (e.g. PARPi monotherapy alone). Yet, this should be extensively tested in the future. For the present, it will be interesting to understand if the same model can be applied to different available therapies, for example to evaluate the opportunity to add carboplatin to standard anthracycline-taxane-based neoadjuvant chemotherapy, to avoid the cardiotoxic anthracyclines or even the evaluate the addition of immune-checkpoint inhibitor pembrolizumab. A better correlation with clinical outcomes has to be further tested, as well.

Another limitation, is that SUV_max_ detected by ^18^FDG-PET/CT is not the standard of care for assessing response to neoadjuvant therapy in BC. However, recent results of the PHERGain trial in early-stage HER2 + BC, showed that ~ 1/3 of patients with HER2 + BC treated with neoadjuvant trastuzumab and pertuzumab might be spared chemotherapy, thanks to the degree of metabolic response detected with FDG-PET/CT after just 2 cycles of the anti-HER2 combination and subsequent type of pathologic response detected after surgery^51^. In this line, our study has to be intended as proof-of-concept analysis where our aim was to essentially prove that a PDEs reactive–diffusive mathematical model based on the assumption of Gompertzian growth could be applied not only to a setting where tumor dimension/growth is directly considered, but also to demonstrate the capability to track tumor metabolism changes as detected by a standardized and objective measurement parameter (i.e. ^18^FDG-PET SUV_max_) as a surrogate of malignancy and relate it to tumor response to treatment. In this perspective, we believe we succeeded in our intent, though replicating our findings in other independent but similar databases will be crucial. Finally, the software COMSOL Multiphysics is not open-access and a more user-friendly interface should be envisioned for a broader implementation outside a pure Engineer/Mathematic environment. Nevertheless, COMSOL’s computational robustness is already consolidated and the proposed mathematical model has the advantage of potentially being run at low cost on standard desktop computers, being also virtually adaptable to any proliferation/therapy scenarios, as also preliminarily observed in Lymphomas and a different BC setting^17,21^.

To conclude, we observed that a mathematical framework based on realistic multidimensional governing PDEs, could be directly applied to the tailored simulation of an early therapeutic response to the PARPi olaparib in early-stage TNBC by using ^18^FDG-PET/CT scan, at the single patient level. The analytical and computational structure of the model sets the basis for further development in *in-silico* prognosis in Oncology, where the model has the potential to be tested in any virtual scenario on any possible patient, for any combination of the variable space in a sustainable way, to inform and support the Oncologists in their therapeutic decisions.

Prospective evaluation in independent cohorts and correlation of these outcomes with more recognized efficacy endpoints is now warranted.

## Data Availability

The datasets generated during and/or analyzed during the current study are available from the Corresponding Author upon reasonable request.

## Abbreviations

BC: Breast cancer
CAFs: Cancer-associated fibroblasts
CI: Confidence interval
CP: Challenged proliferation
CT: Chemotherapy
ECM: Extra-cellular matrix
ESMO: European society for medical oncology
ε_PD_: Nominal personalized drug efficiency
FP: Free proliferation
gBRCA: Germline BRCA1/2
PDEs: Partial differential equations
NAT: Neoadjuvant therapy
PARP inhibitor: PARPi
PD: Pharmacodynamics
PK: Pharmacokinetic
ROI: Region of interest
SD/PD: Stable disease/progressive disease
SUV_max_: Maximum standard uptake value
SUV_max_*: Predicted SUV_max_ values in silico
*t*: Time
*t*i: Indefinite timepoint
*t*0: Baseline timepoint
TILs: Tumor-infiltrating lymphocytes
TNBC: Triple negative breast cancer
